# Strong impact of sulfotransferases on DNA adduct formation by 4‐aminobiphenyl in bladder and liver in mice

**DOI:** 10.1002/cam4.1779

**Published:** 2018-10-10

**Authors:** Yun Li, Zhidan Chen, Joseph D. Paonessa, Walter Meinl, Arup Bhattacharya, Hansruedi Glatt, Paul Vouros, Yuesheng Zhang

**Affiliations:** ^1^ Department of Pharmacology and Therapeutics Roswell Park Comprehensive Cancer Center Buffalo New York; ^2^ Department of Urology Roswell Park Comprehensive Cancer Center Buffalo New York; ^3^ Barnett Institute and Department of Chemistry and Chemical Biology Northeastern University Boston Massachusetts; ^4^ Department of Molecular Toxicology German Institute of Human Nutrition Potsdam‐Rehbruecke (DIfE) Nuthetal Germany; ^5^ Department of Food Safety German Federal Institute for Risk Assessment (BfR) Berlin Germany; ^6^ Department of Cancer Prevention and Control Roswell Park Comprehensive Cancer Center Buffalo New York; ^7^ Present address: Shire, 300 Shire Way Lexington Massachusetts

**Keywords:** 4‐Aminobiphenyl, bladder cancer, gender‐related risk of bladder cancer, sulfotransferase, tobacco carcinogen

## Abstract

Bladder cancer risk is 3‐4 times higher in men than women, but the reason is poorly understood. In mice, male bladder is also more susceptible than female bladder to 4‐aminobiphenyl (ABP), a major human bladder carcinogen; however, female liver is more susceptible than male liver to ABP. We investigated the role of sulfotransferase (Sult) in gender‐related bladder and liver susceptibility to ABP. Sulfation reactions of aromatic amine bladder carcinogens catalyzed by Sult may generate highly unstable and toxic metabolites. Therefore, liver Sult may decrease bladder exposure to carcinogens by promoting their toxic reactions in the liver. Notably, the expression of several liver Sults is suppressed by androgen in male mice. Here, we show that two Sults are critical for gender‐related bladder susceptibility to ABP in mice. We measured tissue level of *N*‐(deoxyguanosin‐8‐yl)‐4‐aminobiphenyl (dG‐C8‐ABP), a principal ABP‐DNA adduct, as readout of tissue susceptibility to ABP. We identified Sutl1a1 and to a lesser extent Sult1d1 as Sults that promote dG‐C8‐ABP formation in hepatic cells. In mice, gender gap in bladder susceptibility to ABP was narrowed by knocking out Sult1a1 and was almost totally eliminated by knocking out both Sutl1a1 and Sult1d1. This was accompanied by dramatic decrease in ABP genotoxicity in the liver (>97%). These results show the strong impact of the Sults on bladder and liver susceptibility to a human carcinogen. Because liver expression of both Sult1a1 and Sutl1d1 is suppressed by androgen in male mice, our results suggest that androgen renders bladder more exposed to ABP in male mice by suppressing Sult‐mediated ABP metabolism in liver, which increases bladder delivery of carcinogenic metabolites.

## 1 INTRODUCTION

Men have 3‐4 times higher risk of developing bladder cancer (BC) than do women.^1^, ^2^, ^3^ The reason for the increased BC risk in men is poorly understood. Tobacco smoking is the most important cause of BC; population attributable risk for tobacco smoking in BC is approximately 50% in both men and women.^2^ However, gender disparity in BC risk exists in both smokers and nonsmokers.^2^, ^3^ Aromatic amines are the main bladder carcinogens in tobacco smoke, but nonsmokers are also exposed to these carcinogens through environmental and occupational contact.^4^, ^5^ While androgen and androgen receptor may promote bladder cell proliferation and bladder tumorigenesis,^6^, ^7^ there is also evidence that sex hormones may influence bladder cancer risk by modulating carcinogen metabolism in liver and other organs.^8^

4‐Aminobiphenyl (ABP) is a major human bladder carcinogen in tobacco smoke.^9^ Levels of ABP‐DNA adducts are up to eightfold higher in bladder specimens or exfoliated urothelial cells of smokers than of nonsmokers.^10^, ^11^ Approximately 80% of ABP‐DNA adducts formed in bladder cells and tissues are *N*‐(deoxyguanosin‐8‐yl)‐4‐aminobiphenyl (dG‐C8‐ABP).^12^ In BALB/c mice exposed to ABP in drinking water for 4 weeks, dG‐C8‐ABP levels are twofold to threefold higher in the male bladder than in the female bladder, but levels of this adduct are twofold to threefold higher in the female livers than in the male livers.^13^ Moreover, in BALB/c mice exposed to ABP in drinking water for 96 weeks, BC developed in 20% of the male mice but in none of the female mice, whereas liver tumor developed in 33% of the female mice but in none of the male mice.^14^ We also found that in C57BL/6 mice, at 24 hours after treatment with a single dose of ABP, bladder dG‐C8‐ABP level is 3.1‐fold higher in the male than in the female, but liver dG‐C8‐ABP is 4.8‐fold higher in the female than in the male.^15^ We further showed that castration causes male mice to acquire the female phenotype in dG‐C8‐ABP formation in bladder and liver, while spaying female mice has little effect.^15^ Given that ABP and other aromatic amine carcinogens are metabolized mainly in the liver, the above findings suggest that androgen may cause the dichotomy of carcinogenicity of aromatic amines in the bladder and liver by modulating certain liver metabolic enzymes.

Liver metabolism of ABP and other aromatic amines include hydroxylation catalyzed by cytochrome p450 enzyme (CYP), acetylation catalyzed by arylamine acetyltransferase (NAT), glucuronidation catalyzed by UDP‐glucuronosyltransferase (UGT), and sulfation catalyzed by sulfotransferase (SULT).^8^ *N*‐hydroxylation is considered the first step in activation of aromatic amine carcinogens. However, total liver *N*‐hydroxylation activity toward ABP is not different between male and female C57BL/6 mice (Figure S1). Moreover, while CYP1A2 was thought to be mainly responsible for *N*‐hydroxylation of aromatic amines, knockout (KO) of this enzyme in C57BL/6 mice did not cause a significant impact on ABP‐DNA adduct formation in the liver and bladder.^16^ Two NATs participate in the metabolism of aromatic amines. However, in C57BL/6 mice or other mice, liver NAT activity toward ABP does not show a gender disparity, and in congenic mouse strains of rapid and slow acetylators, the acetylation status did not show a significant impact on ABP‐DNA adduct formation in both bladder and liver.^17^ Liver UGT catalyzes the conjugation of aromatic amines with glucuronic acid; the conjugates are excreted in the urine and are labile, delivering carcinogenic metabolites to the bladder.^8^ Indeed, we showed that transgenic mice with liver expression of an ABP‐metabolizing human UGT have increased dG‐C8‐ABP level in the bladder following exposure to ABP.^15^ However, in wild‐type (WT) mice (C57BL/6), liver ABP‐specific UGT activity is significantly higher in female mice than in male mice, and in castrated male mice, liver UGT activity is similar to that in female mice.^15^ These results suggest that CYP, NAT, and UGT in liver may not play a significant role in the gender‐specific bladder susceptibility to ABP.

Sulfotransferase catalyzes the sulfation of aromatic amine metabolites, which generates highly unstable and toxic metabolites.^8^ It was previously shown that liver Sult activity correlates with liver toxicity and liver tumor development in rats treated by *N*‐hydroxy‐2‐acetylaminofluorene, a metabolite of 2‐aminofluorene, which is also an aromatic amine.^18^ It was hypothesized that the sulfuric acid esters of aromatic amines generated in the liver, while toxic to liver, might be too labile to survive the trip to the bladder.^19^ However, liver SULT may alter the bioavailability of aromatic amine metabolites to the bladder by competing with other liver enzymes for biotransformation of the compounds. The mRNA levels of multiple hepatic Sult isoforms are expressed 2‐ to 100‐fold higher in female mice than male mice, including Sult1a1, Sult1c2, Sult1d1, Sult2a1/a2, and Sult3a1, and the gender difference in their expression level is eliminated by castration but not spaying,^20^, ^21^ showing that androgen suppresses their expression in the liver.

## 2 MATERIALS AND METHODS

### 2.1 Chemicals

ABP was purchased from Sigma‐Aldrich (St. Louis, MO, USA). *N*‐hydroxy‐4‐aminobiphenyl (*N*‐OH‐ABP) was purchased from Toronto Research Chemicals (North York, Ontario, Canada).

### 2.2 Cell culture, gene transfection, and treatment with *N*‐OH‐ABP

Mouse hepatic cell line Hepa1c1c7 (ATCC, Manassas, VA, USA) was cultured in αMEM supplemented with glutamine and 10% fetal bovine serum in a humidified incubator with 5% CO_2_. Mouse Sult isoforms were expressed in Hapa1c1c7 cells by transient gene transfection. The Sult expression plasmids were purchased from Origene (Rockville, MD, USA), including Sult1c2 (MR204091), Sult2a1 (MR221316), Sult2a2 (MR225420), Sult3a1 (MR220047), and Sult1d1 (MR204074). Sult1a1 plasmid was generated in our own laboratory. The full‐length mouse Sult1a1 coding sequence (GenBank NM_133670.2) was amplified by PCR using SgfI‐forward primer (5′‐GCGATCGCCatggctcagaaccccagc‐3′) and MIuI‐reverse primer (5′‐ACGCGTccctatttgacagcg gaacg‐3′). The amplified PCR product was digested by SgfI and MIuI (Thermo Fisher Scientific, Waltham, WA, USA) and ligated into pCMV6‐AC‐Myc‐DDK (Origene) which was predigested with the same restriction enzymes. The insert was confirmed by DNA sequencing.

For gene transfection, cells were cultured in 6‐well plates (0.2 × 10^6^ cells/well) overnight, transfected with a Sult plasmid or empty vector (EV; 2 μg DNA per well) using Lipofectamine 2000 (Thermo Fisher Scientific), and harvested 24 hours after plasmid transfection. To determine the effect of a specific Sult on DNA adduct formation induced by *N*‐OH‐ABP, Hepa1c1c7 cells were transfected with a Sult expression plasmid or the empty vector for 24 hours and then treated with solvent or *N*‐OH‐ABP. The cells were harvested by trypsin treatment and centrifugation and washed once with phosphate‐buffered saline, typically pooling cells from 3 to 4 wells into one pellet.

### 2.3 Western blotting

Cells were lysed by sonication (Branson Model 450 sonifier) in ice‐cold 50 mmol/L potassium phosphate buffer (pH 7.4) after suspending each cell pellet described above in 0.1 mL buffer. The cell lysates were cleared by centrifugation at 9000 *g* for 20 minutes at 4°C, quantified for protein content by Pierce BCA assay kit, and measured for Sult expression by Western blot analysis. Briefly, samples were mixed with 4x loading dye, heated for 5 minutes at 95°C, and resolved by sodium dodecyl sulfate polyacrylamide gel electrophoresis, followed by transfer to polyvinylidene difluoride membrane. Proteins on the membrane were probed with specific antibodies and detected using Luminata Classico (Millipore, Burlington, MA, USA). All Sult isoforms were detected using an antibody for the DDK tag (Origene, cat # TA50011).

### 2.4 Measurement of Sult activity

Cell samples were prepared as described above under Western blotting. Sult activity in cell samples was measured using *N*‐OH‐APB as a substrate, following published procedures^22^, ^23^ with minor modification. Briefly, enzymatic activity was measured in 60 μL reaction solution in a glass vial, to which a sample (60 μg protein) and 3′‐phosphoadenosine‐5′‐phosphosulfate (PAPS; 100 μmol/L, final) were added in 58 μL of 50 mmol/L potassium phosphate buffer (pH 7.4), and the substrate (100 μmol/L final) was added in 2 μL of methanol. Each reaction was carried out in a 37°C water bath for 5‐10 minutes and stopped by addition of 100 μL of an ice‐cold solution consisting 80% methanol and 20% 50 mmol/L potassium phosphate buffer (pH 7.4). The reaction solutions were immediately centrifuged (16 000 *g*) for 4 minutes at −4°C, and the supernatant fraction was promptly stored at −80°C until measurement by high‐performance liquid chromatography (HPLC) for 3′‐phosphoadenosine‐5′‐phosphate (PAP).

HPLC measurement of PAP in a sample was carried out using an Agilent system (1100 series). Typically, 40 μL sample was loaded to an analytical reverse‐phase Partisil 10 ODS‐2 column (Hichrom, Berkshire, UK), which was eluted with an isocratic mobile phase consisting of 7% methanol and 93% of phosphate buffer containing 75 mmol/L potassium dihydrogen phosphate, 100 mmol/L ammonium chloride, and 1 mmol/L octylamine at a flow rate of 1.75 mL/min, with the detection wavelength set at 260 nm. PAP is eluted approximately at 18 minutes (Figure S2), and its amount was calculated based on comparison with a PAP standard.

### 2.5 Mice and ABP treatment

FVB/N mice (WT) were purchased from Envigo (Frederick, MD, USA) and were acclimated for 1 week before use. Mice with Sult1a1 KO and KO of Sult1a1 and Sult1d1 in FVB/N background were bred in our own laboratory by mating male and female homozygous KO mice. Construction of the KO mice has been previously described.^24^ Briefly, exons 2‐4 of the *Sult1a1* gene or *Sutl1d1* gene were replaced with a neomycin resistance cassette by homologous recombination. We confirmed gene knockout in the mice used in the present study by PCR genotyping (Appendix S1). Mice (8‐9 weeks of age) were treated with a single dose of vehicle or ABP (20 mg/kg body weight) or ABP (2 mg/kg) once daily for 7 days by intraperitoneal injection (i.p.). ABP was dissolved in dimethyl sulfoxide and was given to mice in a volume of 2.5 μL/g body weight. The mice were killed 24 hours after final treatment, and their bladder and liver were removed for analysis. The animal protocols were approved by the Roswell Park Comprehensive Cancer Center Animal Care and Use Committee.

### 2.6 Measurement of dG‐C8‐ABP

Sample preparation (DNA purification from cells and tissues as well as DNA hydrolysis) and measurement of dG‐C8‐ABP by capillary liquid chromatography and nanoelectrospray ionization‐tandem mass spectrometry (LC/MS/MS) have been previously described.^25^

### 2.7 Statistical analysis

Student's *t* test and analysis of variance were used for two‐group and multigroup comparisons (followed by Tukey multiple comparisons test), respectively. *P* value of 0.05 or lower was considered statistically significant.

## 3 RESULTS

### 3.1 Sult1a1 and Sult1d1 promote dG‐C8‐ABP formation in hepatic cells

**Figure 1:**
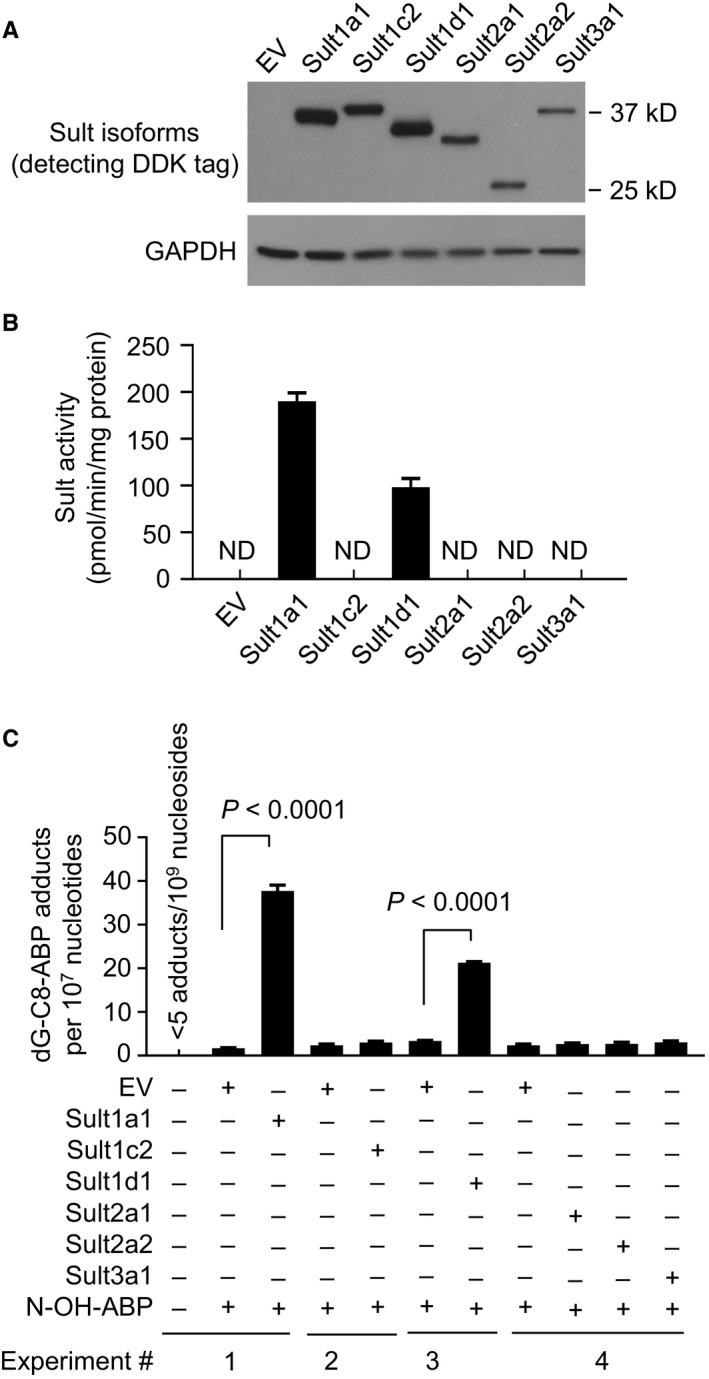
The expression of Sult isoforms, their catalytic activities toward *N*‐OH‐ABP, and their effects on DNA adduct formation in mouse hepatic cells exposed to *N*‐OH‐ABP. Hepa1c1c7 cells were transfected with a plasmid expressing a specific Sult isoform or EV; 24 h later, the cells were either harvested for measurement of expression of each Sult isoform by Western blotting (A) and Sult enzymatic activity using *N*‐OH‐ABP as the substrate (B) or treated with *N*‐OH‐ABP at 30 μmol/L for 3 h, followed by measurement of dG‐C8‐ABP by LC/MS/MS (C). Each value in B and C is a mean ± SEM (n = 3)

We first measured the expression of each mouse Sult and their enzymatic activity toward *N*‐OH‐ABP. Mouse hepatic Hepa1c1c7 cells were transfected with a plasmid with or without expressing a specific Sult for 24 hours, from which whole cell lysates were prepared, measured for Sult protein expression, and analyzed for its enzymatic activity toward *N*‐OH‐ABP. Significant expression of each Sult was detected by Western blotting, although their expression levels varied to some extent (Figure 1A). Lysates of cells transfected with EV, Sult1c2, Sult2a1, Sult2a2, or Sult3a1 showed no catalytic activity, whereas significant catalytic activity was detected in lysates with Sult1a1 or Sult1d1 (Figure 1B). Sult1a1 was nearly twice as active as Sult1d1. We next measured the effect of each of the aforementioned mouse Sults on formation of dG‐C8‐ABP in Hepa1c1c7 cells. Cells were transfected with EV or a specific Sult for 24 hours and then treated with *N*‐OH‐ABP (30 μmol/L, 3 hours). *N*‐OH‐ABP is the starting metabolite in ABP bioactivation. The *N*‐OH‐ABP treatment condition was based on a preliminary dose‐ and time‐finding experiment. The purpose of the experiments was to identify any Sult that might potentiate dG‐C8‐ABP formation. A relatively high concentration of *N*‐OH‐ABP was used, so as not to miss any Sult that might be relatively weak in potentiating adduct formation. dG‐C8‐ABP was measured by LC/MS/MS and was undetectable in untreated Hepa1c1c7 cells. Each Sult was significantly expressed in Hepa1c1c7 cells as described above, but only Sult1a1 and Sult1d1 activated *N*‐OH‐ABP, increasing dG‐C8‐ABP level 22.3‐ and 6.4‐fold, respectively (Figure 1C). This result is consistent with the catalytic activity of each Sult toward *N*‐OH‐ABP. Notably, no dG‐C8‐ABP was detected in Hepa1c1c7 cells treated with ABP up to 1 mmol/L for 24 hours, apparently due to lack of relevant enzymes to convert ABP to *N*‐OH‐ABP.

### 3.2 KO of Sult1a1 and Sult1d1 protects liver against ABP and erases gender‐related bladder susceptibility to ABP

**Figure 2:**
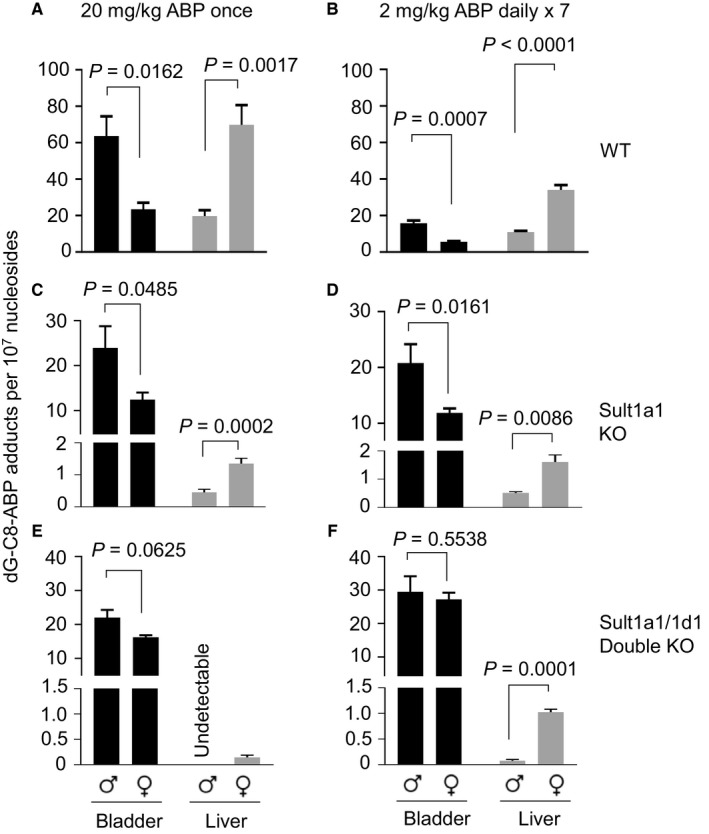
dG‐C8‐ABP formation in the bladders and livers of WT mice, mice with Sult1a1 KO, or mice with KO of both Sult1a1 and Sult1d1. (A, C, E) Mice (8‐9 wk of age) were given a single dose of ABP (20 mg/kg, i.p.); 24 h later, the bladders and livers were collected for measurement of dG‐C8‐ABP level by LC/MS/MS. (B, D, F) Mice (8‐9 wk of age) were given ABP (2 mg/kg, i.p.) once daily for 7 d; 24 h after the final dose, the bladders and livers were collected for measurement of dG‐C8‐ABP level by LC/MS/MS. Each value is a mean ± SEM (n = 3‐9)

We next assessed the impact of Sult1a1 and Sult1d1 on dG‐C8‐ABP formation in vivo, using mice with Sult1a1 KO or KO of both Sult1a1 and Sult1d1, along with WT mice, all in FVBN background (Figure S3). Mice of 8‐9 weeks of age were treated with ABP once (20 mg/kg) or over 7 days (2 mg/kg daily), and dG‐C8‐ABP level in bladder and liver was measured at 24 hours after the final treatment, using LC/MS/MS. In WT mice, regardless of ABP dosing schedule, dG‐C8‐ABP level was 2.7‐ to 2.8‐fold higher in the male bladder than female bladder but was 3.1‐ to 3.5‐fold higher in the female liver than male liver (Figure 2A,B). Similar results were shown in BALB/c mice and C57BL/6 mice.^13^, ^15^ Sult1a1 KO caused marked decrease in liver dG‐C8‐ABP level, decreasing 21.2‐ to 21.4‐fold (male‐female) in the single ABP treatment and 51.7‐ to 52.2‐fold (male‐female) in the 7‐day treatment with ABP (Figure 2C,D). Thus, Sult1a1 is the critical ABP activator in liver. However, despite marked decrease in liver dG‐C8‐ABP in mice with Sult1a1 KO, it was still 2.6‐ to 3.0‐fold higher in the female liver than male liver, suggesting minor involvement of other factors (potentially another Sult) in differential sensitivity of male and female livers to ABP. The gap in bladder dG‐C8‐ABP level between male and female mice with Sult1a1 KO narrowed to 1.7‐ to 1.8‐fold whether the mice were treated by ABP in a single dose or for 7 days (Figure 2C,D). However, the change in absolute level of dG‐C8‐ABP in the bladders of Sult1a1 KO mice was not unidirectional; it decreased 1.9‐ to 2.7‐fold (male‐female, *P* < 0.05) in the single ABP treatment but increased 1.3‐ to 2.1‐fold (male‐female, *P* < 0.05) in the 7‐day treatment with ABP, the reason for which is unknown. As expected, liver dG‐C8‐ABP level in mice with KO of both Sult1a1 and Sult1d1 was lower than in mice with only Sult1a1 KO, but it was still relatively higher in female mice than male mice (Figure 2E,F). As in Sult1a1 KO, the double KO mice showed decrease in bladder dG‐C8‐ABP level in the single ABP treatment but increase in the 7‐day treatment with ABP, compared to WT mice. Most interestingly, however, KO of both Sult1a1 and Sult1d1 narrowed the gender gap in bladder dG‐C8‐ABP level to 1.4‐fold in mice treated with the single dose of ABP and to 1.1‐fold in mice treated with ABP over 7 days, and the differences are no longer statistically significant. Thus, Sult1a1 and Sult1d1 are mainly responsible for gender‐specific bladder susceptibility to ABP in mice.

## 4 DISCUSSION

Sults may generate highly reactive and unstable ABP metabolites. Using expression vectors for individual Sult forms, we identified Sult1a1 and to a lesser extent Sult1d1 as Sults that promote dG‐C8‐ABP formation in cultured hepatic cells. Liver expression of both Sult1a1 and Sult1d1 is significantly lower in male mice than female mice due to suppression by androgen.^21^ Higher expression of these enzymes in female liver may explain the higher susceptibility of females to the hepatocarcinogenicity of ABP, compared to the males. We showed that KO of Sult1a1 and Sult1d1 blunted almost completely the formation of liver dG‐C8‐ABP adducts, indicating that other metabolic enzymes such as Nat do not contribute quantitatively to liver toxicity of ABP. These findings also explain that Nat rapid acetylator and slow acetylator showed no significant difference in ABP‐derived liver DNA adducts.^17^

Excess male bladder susceptibility to ABP was markedly reduced in Sult1a1 KO mice and was almost completely eliminated in mice with KO of both Sutl1a1 and Sult1d1. This together with the liver result discussed above suggests that these liver Sults decrease the bioavailability of genotoxic ABP metabolites to the bladder by generating unstable metabolites that are reacted locally. However, besides liver, Sults in other organs may also influence bladder exposure to ABP and its metabolites. For example, Sult1a1 in kidney is also suppressed by androgen.^21^ To confirm the role of a specific Sult in liver or another organ in gender disparity of bladder susceptibility to ABP and other carcinogens, it will be necessary to evaluate mice with organ‐specific deletion or overexpression of the Sult. For this reason, we have not compared the difference in ABP‐induced bladder tumorigenesis in the current study.

Notably, ABP was administered to mice at 20 mg/kg once or 2 mg/kg once daily for 7 days in our study. Humans are exposed to ABP at much lower levels. However, it was not feasible to lower the ABP dose in our study, even though the LC/MS/MS used was highly sensitive for detection of dG‐C8‐ABP, because dG‐C8‐ABP level was extremely low in the livers of Sult KO mice. In fact, liver dG‐C8‐ABP level was undetectable in the Sult1a1 and Sult1d1 double KO mice treated with ABP once at 20 mg/kg (Figure 2E). Other investigators have used similar ABP doses in published studies. For example, Tsuneoka et al^16^ treated mice with ABP at 1‐25 mg/kg in a study focused on the role of cytochrome 1a2 in ABP‐induced liver and bladder DNA damage. Nevertheless, dG‐C8‐ABP measured in our experiments is the main ABP‐DNA adduct formed in humans as mentioned before.

It remains to be assessed for the significance of our findings in mice to humans. SULT1D1 is a pseudogene in human, but SULT1A1 is the major SULT enzyme in human liver.^26^ It is not known that SULT1A1 in human liver is regulated by androgen, but several other human SULTs, besides SULT1A1, also catalyze the sulfation of *N*‐OH‐ABP, such as SULT1A2, SULT1A3, and SULT1C2.^27^ Given the present results, it will be important to investigate whether any human SULT that participates in the metabolism of bladder carcinogens is expressed in the liver in a gender‐related manner. Notably, liver cancer incidence is significantly higher in men than in women.^1^ However, the main risk factors of liver cancer in human, including chronic viral hepatitis, cirrhosis, and exposure to aflatoxins, may mask the potential carcinogenic effects of aromatic amines.

## ACKNOWLEDGMENTS

We thank Dr. Stephanie Krämer (German Institute of Human Nutrition Potsdam‐Rehbrücke) and Robyn B. Wilkins (Roswell Park Comprehensive Cancer Center) for research assistance.

## CONFLICT OF INTEREST

None.
