# Supplementary figures and images for: Strong impact of sulfotransferases on DNA adduct formation by 4‐aminobiphenyl in bladder and liver in mice
Source: Cancer Med. 2018 Oct 10;7(11):5604–10. doi: 10.1002/cam4.1779 (PMC6246946; doi:10.1002/cam4.1779)

A

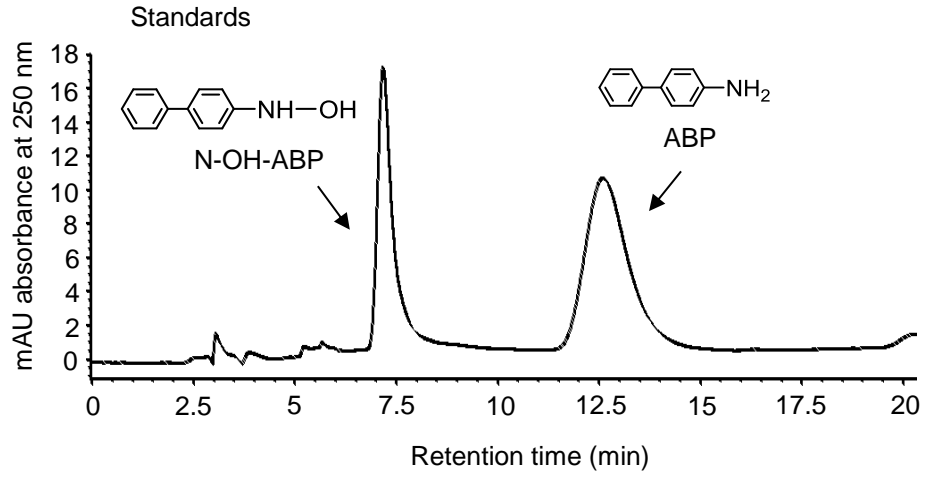

B

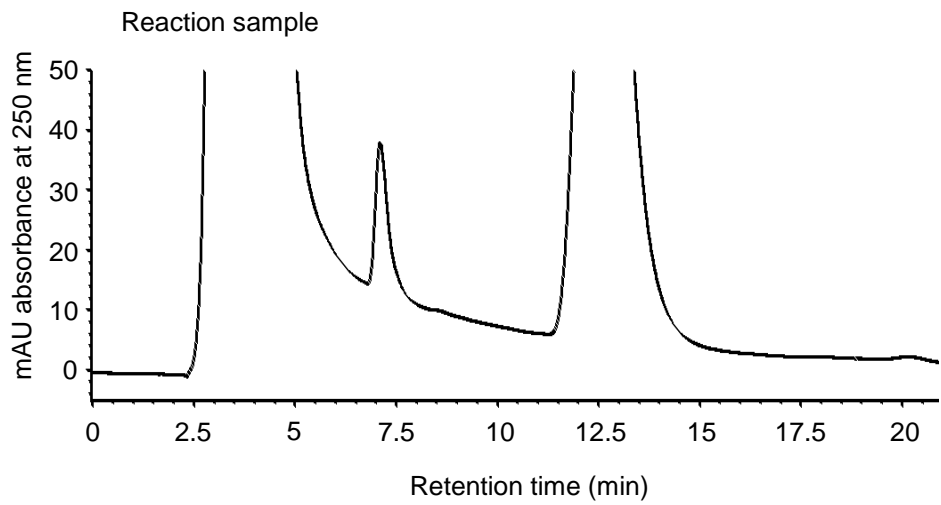

C

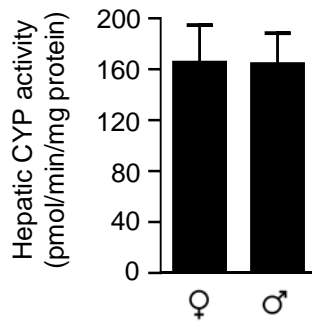

Supplement: Supplementary file 1 [file CAM4-7-5604-s001.pdf]

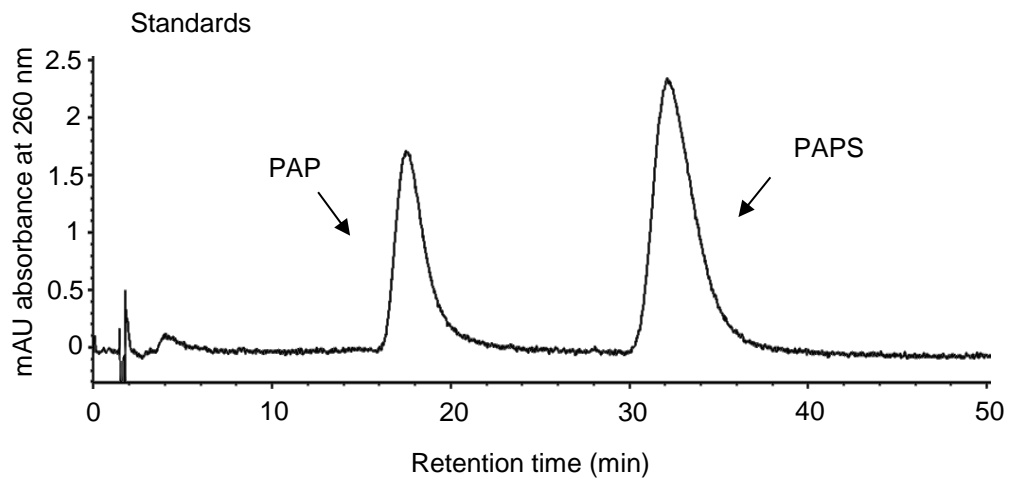

Supplement: Supplementary file 2 [file CAM4-7-5604-s002.pdf]

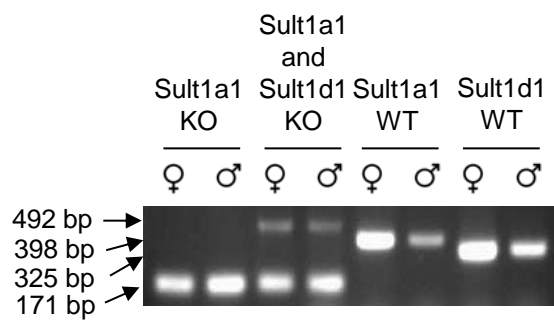

Supplement: Supplementary file 3 [file CAM4-7-5604-s003.pdf]
