# Supplementary material for: Strong impact of sulfotransferases on DNA adduct formation by 4‐aminobiphenyl in bladder and liver in mice
Source: Cancer Med. 2018 Oct 10;7(11):5604–10. doi: 10.1002/cam4.1779 (PMC6246946; doi:10.1002/cam4.1779)
Supplement: Supplementary file 4 [file CAM4-7-5604-s004.docx]

**Supplemental Information**

**Materials and Methods**

**Chemicals**

NADP+ was purchased from Amresco (Solon, OH). Glucose-6-phosphate and glucose-6-phosphate dehydrogenase were purchased from Sigma-Aldrich (St. Louis, MO).

**Genotyping of mice**

Sult1a1 KO was confirmed, using primers for the inserted neomycin cassette: forward 5'-TGAATGAACTGCAGGACGAG-3' and reverse 5'-ATACTTTCTCGGCAGGAGCA-3', which generates a 171 bp fragment. Sult1d1 KO was confirmed, using primers for the inserted neomycin cassette: forward 5'-TGGACTGATAGCCCCATTTC-3' and reverse 5'-GGGGAACTTCCTGACTAGGG-3', which generates a 492 bp fragment. For comparison, the following primers were also used to detect the WT genes: for Sult1a1 (exons 3 and 4), forward 5'-CACACACTTTGGTAAGCAGGTC-3' and reverse 5'-GGGTTTTCTGTTAGTTGGTTGG-3', which generates a 398 bp fragment, and for Sult1d1 (exon 4), forward 5'-CAGATGCGGAAATCATTGTG, and reverse 5'-GAAGCAGCTGAACAGGAAGG, which generates a 325 bp fragment.

**Measurement of hepatic CYP activity**

C57BL/6 mice (7-8 weeks of age), purchased from Tocanic (Rensselaer, NY), were killed, and livers were immediately collected. The livers were homogenized in ice-cold 50 mM potassium phosphate buffer (pH 7.4), using Kontes Dual 22 glass tissue grinders connected to an electric homogenizer. The homogenates were centrifuged at 9,000 g at 4 ^o^C for 20 min. The supernatant fractions were further centrifuged at 126,000 g for 1 h at 4 ^o^C to pellet microsomes. Microsomal pellets were resuspended in up to 0.5 ml of the same buffer for measurement of CYP enzyme activity. Protein concentration of each sample was determined using the BCA assay.

CYP activity towards ABP was measured following a published protocol [^1^](#_ENREF_1) with minor modifications. Briefly, each 0.2 ml reaction was carried out in 50 mM potassium phosphate (pH 7.4), containing microsomes (0.75 mg protein), 0.5 mM ABP (dissolved in methanol, 2% methanol in reaction solution), and an NADPH generating system (0.5 mM NADP+, 5 mM glucose-6-phsophate, 2 units of glucose-6-phosphate dehydrogenase). The NADPH generating system was pre-incubated at 37 ^o^C for 1 min before being added to the reaction solution to initiate the enzymatic reaction. The reaction was performed in a glass vial for 10 min at 37 ^o^C and terminated by addition of 0.2 ml of ice-cold acetonitrile. The solutions were then centrifuged at 16,000 g for 4 min at -4 ^o^C, and the supernatants were immediately stored in -80 ^o^C freezer until analysis by HPLC in order to minimize *N*-OH-ABP degradation.

HPLC measurement of *N*-OH-ABP as well as ABP in a sample was carried out using an Agilent system (1100 series). Typically, 100 μl sample was loaded to an analytical reverse-phase Partisil 10 ODS-2 column (Hichrom), which was eluted with an isocratic mobile phase consisting of 60% acetonitrile in water containing 0.1% acetic acid at a flow rate of 0.75 ml/min. The eluates were monitored at 254 nm by a photodiode array detector. *N*-OH-ABP and ABP were eluted at approximately at 7.5 min and 12.5 min, respectively (Figure S1), and the amount of *N*-OH-ABP via peak area integration was calculated based on comparison with *N*-OH-ABP standards.

**Reference**

1. Nakajima M, Itoh M, Sakai H, Fukami T, Katoh M, Yamazaki H, Kadlubar FF, Imaoka S, Funae Y, Yokoi T. (2006). CYP2A13 expressed in human bladder metabolically activates 4-aminobiphenyl. *Int J Cancer* 119, 2520-2526.

**Figure legend**

**Figure S1.** Measurement of CYP activity in mouse liver. (*A*) HPLC of *N*-OH-ABP and ABP standards. (*B*) HPLC of a representative reaction sample. (*C*) ABP-specific hepatic CYP activity in male and female mice. Each value is a mean ± SD (n=3).

**Figure S2.** HPLC retention times of PAP and PAPS.

**Figure S3.** PCR genotyping of mice, including WT mice, Sult1a1 KO mice, and mice with KO of both Sult1a1 and Sult1d1.
